# A Precautionary Tale: Mental Health and Risk Communication

**Published:** 2005-04

**Authors:** Bob Weinhold

Many agencies and organizations have, over the years, assumed that a substance should be assumed benign until proven harmful, and that caution is needed only in limited cases. Advocates of the pre-cautionary principle, on the other hand, assume that it is better to be cautious in the face of scientific uncertainty about a potential threat—such as electromagnetic fields (EMFs) from cellular phones and cell phone towers—which should be presumed to be harmful until proven otherwise. However, one important component of overall health, mental health, might not benefit from following the pre-cautionary principle, according to a small European study **[*****EHP*** **113:402–405]**.

To observe how following the precautionary principle affects certain facets of mental health, researchers at the Research Centre Jülich, a German organization funded primarily by the country’s federal government, surveyed students and employees at Austria’s University of Innsbruck and evaluated their responses to precautionary statements. The surveys focused on the issue of EMFs (which the researchers called “electrosmog”) from wireless communications.

In two separate experiments in 2003 and 2004, the researchers presented respondents with various sets of statements about the electrosmog controversy and risk management responses. All the participants saw a base statement noting that there is widespread debate about the potential risks related to electrosmog, although the International Commission on Non-Ionizing Radiation Protection says that current exposure limits adequately protect the public. Some of the participants also saw various additional statements describing measures taken to protect against the potential electrosmog threat, such as “Following a precautionary approach, Switzerland has tightened exposure limits by a factor of 10 in areas where people are exposed for long periods of time.” Statements in the first experiment focused on health-related measures such as stricter exposure limits, while the single additional statement used in the second experiment focused on enhancing public participation in deciding where to build cell phone towers.

After reading the statements, subjects were asked for their perception of the extent of the risk and the quality of the scientific knowledge about electrosmog. In the first experiment respondents who read any of the health precautionary statements felt significantly more threatened than those who read only the base statement. The researchers write that these results support the idea that “precautionary measures will be considered a cue that risk might be real and increase perceived risk.”

This effect was not found in the second experiment, which used a public participation measure. However, respondents who read about public participation had less trust in public health protection.

The team concludes that policy makers should be aware of this downside to taking a precautionary approach, even though enacting such an approach may lead to reduced exposures and improved physical health. However, they acknowledge that more study needs to be done to confirm their results before drawing any practical conclusions for policy making.

## Figures and Tables

**Figure:**
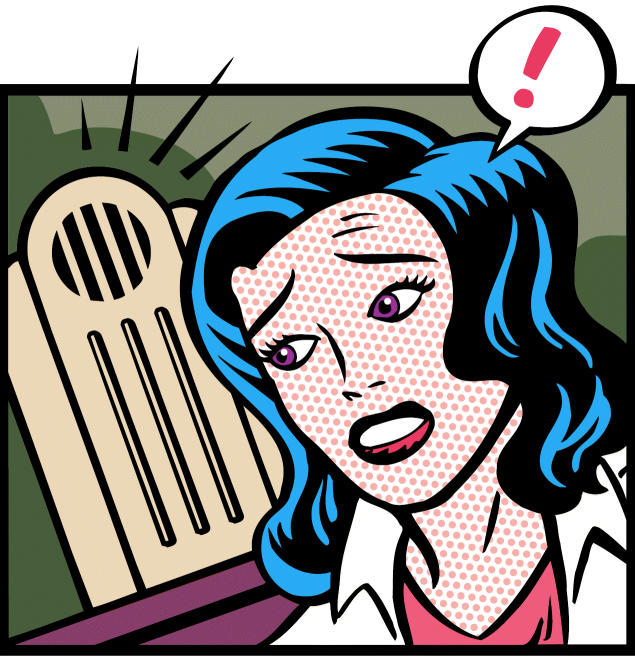
**Is ignorance bliss?** The precautionary principle, which espouses caution in the face of scientific uncertainty, may cause unintended alarm among the public.

